# Artificial intelligence predicts sex-specific risk of metabolic dysfunction-associated steatotic liver disease

**DOI:** 10.1186/s13293-026-00917-6

**Published:** 2026-05-08

**Authors:** Mohamad Jamalinia, Giovanni Targher, Amedeo Lonardo, Seyed Taghi Heydari, Kamran Bagheri Lankarani

**Affiliations:** 1 Gastroenterohepatology Research Center, Shiraz University of Medical Sciences, Shiraz, 7193635899 Iran; 2 Metabolic Diseases Research Unit, IRCCS Sacro Cuore - Don Calabria Hospital, Negrar Di Valpolicella, (VR) Italy; 3 Department of Medicine, University of Verona, Verona, Italy; 4 Department of Internal Medicine, Azienda Ospedaliero-Universitaria Di Modena (-2023), Modena, Italy; 5 Health Policy Research Center, Institute of Health, Shiraz University of Medical Sciences, Shiraz, Iran

**Keywords:** Artificial intelligence, Cardiometabolic risk, Deep learning, Diagnostic performance, Machine learning MASLD, Precision medicine, Predictive modeling, Sex differences

## Abstract

**Background & aims:**

Metabolic dysfunction-associated steatotic liver disease (MASLD) exhibits well-established sex differences across its risk factors, disease progression, and liver-related and extrahepatic outcomes. We trained sex-specific machine learning (ML) algorithms using routine clinical data to evaluate sex-specific learning patterns and diagnostic performance.

**Methods:**

In this cross-sectional study conducted at a cardiology referral center, 446 adults were enrolled. Participants were divided into training (127 men, 185 women) and test sets (55 men, 79 women). Eight ML classifiers were trained on the overall dataset and separately for men and women to predict MASLD presence and steatosis severity (no/mild/moderate-to-severe) as assessed by ultrasonography. Hyperparameters were tuned using grid search cross-validation, and model performance was evaluated on an unseen test set.

**Results:**

The prevalence of MASLD was 63.6% among participants; 41.2% had mild, and 22.4% had moderate-to-severe steatosis. Compared to models trained on the overall dataset, sex-specific modeling improved diagnostic performance in men but remained suboptimal in women. For MASLD presence, top-performing models achieved AUC/F1 scores of 0.769/0.856 overall, 0.793/0.897 in men, and 0.681/0.794 in women. For steatosis severity, respective AUC/F1 scores were 0.761/0.671 overall, 0.723/0.608 in men, and 0.718/0.571 in women. Sensitivity analyses using stratified cross-validation confirmed the performance gap between men and women. Threshold analyses showed acceptable rule-in and rule-out performance in men but suboptimal performance in women. Feature-importance rankings differed substantially between sexes, indicating distinct sex-specific learning patterns.

**Conclusions:**

Artificial intelligence-based algorithms identify sex-specific learning patterns in MASLD steatosis risk prediction. Routine clinical variables appear more informative for men, while showing weaker algorithmic performance in women. This finding suggests that failure to train MASLD risk algorithms with sex-specific risk factors may increase the risk of misclassification in women. Therefore, achieving more equitable and clinically reliable models will require integrating women-specific risk factors and adopting sex-stratified data processing strategies within MASLD prediction frameworks to reduce diagnostic inequities and support more personalized care.

**Supplementary Information:**

The online version contains supplementary material available at 10.1186/s13293-026-00917-6.

## Highlights

Eight machine learning (ML) algorithms were trained on routine clinical data from a cardiology referral center, once using the overall dataset and once separately for men and women. The presence of metabolic dysfunction-associated steatotic liver disease (MASLD) and the severity of steatosis were used as outcomes.

Feature importance rankings revealed significant sex-based differences in algorithm learning, which were masked when training on the overall dataset.

Compared to models trained on the overall dataset, sex-specific modeling improved diagnostic performance in men but remained suboptimal in women, suggesting that routine clinical variables are more informative for men.

Failure to train MASLD risk algorithms with sex-specific risk factors may increase the risk of misclassification among women.

Achieving more equitable and clinically reliable models will require integration of sex-specific risk factors and adoption of sex-stratified data processing in MASLD prediction frameworks to reduce diagnostic inequities and support more personalized care, particularly for women.

**Supplementary Information:**

The online version contains supplementary material available at 10.1186/s13293-026-00917-6.

## Abstract (plain language summary)

Metabolic dysfunction-associated steatotic liver disease (MASLD), formerly known as non-alcoholic fatty liver disease (NAFLD), is the most common liver condition worldwide, affecting approximately one in three adults. Clear differences between men and women have been observed across the MASLD disease course, including its risk factors, prevalence, progression, and multisystem complications. However, most diagnostic tools and machine learning (ML) approaches do not fully account for these sex differences. Sex is typically included as a single variable in models trained on combined datasets, rather than used to develop sex-specific prediction strategies. In this study, we trained, for the first time, separate ML models for men and women and compared their performance with models developed on the overall dataset to investigate whether routine clinical information predicts MASLD equally well for both sexes. Models that included routine clinical measurements performed better in men than in women. Consequently, women may be at higher risk of misclassification when their unique MASLD risk factors are ignored. Our findings highlight the need for sex-specific data processing and modeling approaches to ensure fair, accurate, and equitable MASLD detection for all, with particular focus on improving diagnosis in women.

**Supplementary Information:**

The online version contains supplementary material available at 10.1186/s13293-026-00917-6.

## Introduction

Metabolic dysfunction-associated steatotic liver disease (MASLD), previously known as nonalcoholic fatty liver disease (NAFLD), has become the most common chronic liver disease (CLD) worldwide [1, 2]. It affects approximately one in three adults, and its global prevalence is projected to exceed half of the adult population by 2040 [3]. The MASLD spectrum is broad, ranging from isolated steatosis to metabolic dysfunction-associated steatohepatitis (MASH), advanced fibrosis, and cirrhosis, which, in some cases, may lead to liver-related mortality [4]. The insidious nature of disease makes it particularly difficult to identify MASLD since most individuals with this condition remain asymptomatic while slowly transitioning through disease stages [4]. MASLD is also increasingly recognized as a key contributor to cardiovascular-kidney-metabolic (CKM) health, even in early stages before liver-related symptoms appear [5, 6]. Importantly, MASLD is a highly heterogeneous disease, with substantial variability in metabolic profiles, progression rates, and clinical manifestations across different patient populations, further complicating early detection and risk stratification [7].

Growing evidence suggests that MASLD does not develop or progress the same way in men and women [8]. We recognize that an individual’s biological sex continuously affects metabolic health from early life ages, with differences in chromosomal composition and, subsequently, reproductive hormones across the lifespan [9]. Men accumulate more visceral fat, develop insulin resistance sooner, and have worse cardiometabolic risk profiles than premenopausal women [10]. After menopause, however, these biological advantages of the female sex vanish [11]. Post-menopausal women not only ‘catch up’ to men, but sometimes exhibit higher risk factors for MASLD [11]. Men typically develop MASLD at younger ages than women; however, once the liver disease is established, women are more likely to progress to advanced fibrosis than men [8]. Sex-specific patterns have also been observed in MASLD-related extrahepatic clinical outcomes: women develop MASLD-associated cardiovascular events more frequently than men, and men may experience higher rates of developing MASLD-related chronic kidney disease (CKD) and hepatocellular carcinoma (HCC) than women [12, 13]. Overall, these observations strongly support the idea that, driven by major metabolic and hormonal changes, MASLD trajectories differ between men and women.

Nonetheless, it is widely acknowledged that most non-invasive tests for the diagnosis of MASLD (e.g., the fatty liver index [FLI]) use the same variables and cutoffs for men and women [14, 15], resulting in different diagnostic performance between the sexes [16]. Advanced imaging techniques may provide the best evaluation of hepatic steatosis and fibrosis but are not practical or scalable for population-based screening globally [17]. A simple, pragmatic, and cost-effective solution is urgently needed to address the complex, and potentially non-linear way in which metabolic risk factors interact with sex.

Machine learning (ML) represents a significant advance in this field. Rather than relying on traditional linear models, newer ML techniques, ranging from ensemble-based methods to neural networks, can capture subtle interactions among predictors and reveal distinct patterns that may differ between men and women [18]. While building on existing literature that has examined ML-based methods for MASLD detection, none of these have examined sex as a basis for stratified learning; rather, sex is typically included as a single covariate [19]. This fails to consider the intrinsic, sex-specific biological and metabolic framework [20]. In this pioneering proof-of-concept study, we aimed to evaluate whether learning tailored to the individual’s sex improved predictive performance and altered the importance of clinical variables. To this end, we assessed innovative sex-specific ML algorithms for predicting MASLD and its steatosis severity.

## Methods

### Study setting

This cross-sectional single-center study was conducted between November 2022 and September 2023 et al.-Zahra Cardiology Hospital Center, where a cohort of individuals aged ≥ 30 years was enrolled. Detailed inclusion and exclusion criteria have been previously published [5], and the present study represents an ML analysis of this dataset. Briefly, patients referred for coronary computed tomography angiography (CCTA) were eligible for inclusion. We excluded patients with CLD unrelated to MASLD (e.g., alcohol-related liver disease, viral or autoimmune hepatitis), patients using steatogenic medications, those with significant systemic illness and advanced CKD (estimated glomerular filtration rate [eGFR] < 30 mL/min/1.73 m^2^), or those with significant alcohol consumption (> 30 g/day for men and > 20 g/day for women), HIV infection, pregnancy, a history of cancer, or any established cardiovascular disease.

Collected data included demographic characteristics, anthropometric measurements, and findings from physical examinations. Sedentary behavior and physical activity were assessed using the validated short form of the International Physical Activity Questionnaire (IPAQ) [21]. Laboratory measurements included blood urea nitrogen (BUN) and eGFR, estimated by the Chronic Kidney Disease Epidemiology Collaboration (CKD-EPI) equation [22]. Medical records were reviewed to determine the presence of metabolic dysfunction and current medication use. Postmenopausal status was determined using age ≥ 50 years as a proxy, in accordance with established epidemiological definitions [23].

All participants underwent liver ultrasonography in accordance with the study protocol using a Samsung Accuvix A30 system. These procedures were conducted by a qualified technician who was blinded to clinical and biochemical details of participants. Hepatic steatosis was graded ultrasonographically as follows: (i) no steatosis: no increase in hepatic echogenicity; (ii) mild steatosis: diffusely increased hepatic echogenicity with clear visualization of the diaphragm and portal vein walls; and (iii) moderate to severe steatosis: moderate to marked hepatic echogenicity that obscured the visualization of portal vein branches or the diaphragm [24]. The diagnosis of MASLD was based on hepatic steatosis in combination with at least one of five metabolic dysfunction criteria: increased body mass index (BMI) (≥ 25 kg/m^2^), increased waist circumference (WC) (> 94 cm for men and > 80 cm for women), type 2 diabetes mellitus (T2DM), arterial hypertension (HTN) (blood pressure ≥ 135/85 mmHg or antihypertensive drug treatment), or atherogenic dyslipidemia (high plasma triglycerides or low HDL-cholesterol or lipid-lowering medications). CCTA findings were not included in the present analysis, as the objective was to train ML algorithms using simple clinical data to predict MASLD risk.

### Data preprocessing

The primary endpoint of the study was the prediction of MASLD, assessed as a binary outcome (absence vs. presence of MASLD) and as multiclass endpoint, reflecting steatosis severity (no MASLD vs. MASLD with mild steatosis vs. MASLD with moderate-to-severe steatosis). All other variables served as potential predictors. The study sample was randomly split into training (70%) and testing (30%) sets using stratified sampling to preserve class distributions and minimize imbalance, ensuring balanced representation of each sex while maintaining sufficient observations for model training and independent performance evaluation. This procedure resulted in sex-specific sample sizes of 127/55 men and 185/79 women in the training/testing sets.

Predictors were categorized as either numerical or categorical variables. To assess evidence of skewness among continuous predictors, we initially explored their distributions using visualizations. Since there was no evidence of significant skewness, the StandardScaler (z-score normalization) was used to normalize all continuous variables to a comparable scale.

Categorical variables included binary (e.g., sex) and ordinal variables (e.g., physical activity level). Binary variables were encoded as 0/1, while ordinal variables were encoded as integer values (e.g., 0/1/2 or 0/1/2/3). One-hot encoding was not applied to ordinal variables to preserve the natural ordering of categories and allow the models to capture directional relationships between levels.

As this was a prospective cross-sectional study with predefined data collection forms, the dataset was complete at the time of analysis; therefore, no missing-data imputation was required. Preprocessing was implemented using a ColumnTransformer pipeline to standardize numerical features and preserve categorical features unchanged. This pipeline was embedded within the Pipeline framework to ensure reproducibility and avoid disruptions to the workflow and ML process.

### Model development and hyperparameter tuning

A total of eight ML classifiers were assessed for their capability to predict MASLD presence and steatosis severity. The classifiers were: Random Forest (RF), Support Vector Machine (SVM), Stochastic Gradient Descent (SGD) Classifier, Logistic Regression (LR), K-Nearest Neighbors (KNN), Gradient Boosting Classifier (GBC), Gaussian Naive Bayes (GNB), and Multi-Layer Perceptron (MLP). Each ML classifier was integrated into a pipeline that included the entire preprocessing workflow; this ensured uniform feature transformation and avoided data leakage during training and validation. Hyperparameters for each ML classifier were optimized using GridSearchCV with fivefold stratified cross-validation. Search spaces and tuned parameters are provided in Supplementary Tables 3, 4.

### Performance evaluation

After tuning the hyperparameters, the best version of each model was evaluated on the held-out test set. The model’s performance was assessed based on the area under the receiver operating characteristic curve (AUC), F1 score, sensitivity (recall), specificity, positive predictive value (PPV/precision), and negative predictive value (NPV). For multiclass tasks, the metrics were calculated as weighted averages across classes to account for class imbalance. Model robustness was evaluated using 95% confidence intervals (CIs) extracted from 1,000 bootstrap resamples of the final test-set predictions, with the 2.5th and 97.5th percentiles used to calculate the CIs.

Receiver operating characteristic (ROC) curves were produced for all models using predicted probabilities or decision scores to calculate both the false-positive rate (FPR) and the true-positive rate (TPR). In the multiclass classification case, labels were one-hot encoded, and micro-averaged ROC curves with weighted AUCs were generated.

The best model was characterized by the highest combination of AUC and F1 score, favoring F1 when AUC and F1 disagreed, since F1 score is considered the preferred performance metric in imbalanced settings, combining precision and recall into a single metric. To ensure the robustness of model performance estimates, a sensitivity analysis was also conducted for the best models using stratified fivefold cross-validation. The dataset was divided into five mutually exclusive folds while maintaining the proportional distribution of MASLD within each fold. Each iteration utilized 4 folds (80%) for training and onefold (20%) for validation purposes, such that each participant contributed once to validation and four times to training. The process was repeated across all folds, and model performance was summarized as mean ± standard deviation (SD) of the five-fold evaluation metrics. For the best binary model, classification thresholds were evaluated and reported by normalizing decision scores to [0,1] and calculating sensitivity, specificity, PPV, NPV, and F1 score at 0.01 intervals. The optimal threshold was assigned to the highest Youden’s index, while the “rule-in” and “rule-out” thresholds were based on clinically relevant criteria as follows: rule-in was defined as the threshold with the highest PPV among those with specificity > 90%, whereas rule-out was defined as the highest NPV among those with sensitivity > 90%. Due to the complexity of multiclass tasks with multiple boundaries and dominant classes, threshold analysis was not performed for these classifications.

Feature importance analysis was conducted for the top-performing model. Absolute model coefficients were used for linear models (linear SVM, LR, SGD); impurity-based importance for tree-based models (RF, GBC); permutation importance for MLP and KNN; and normalized mean/variance differences for GaussianNB. Importance scores were presented as bar plots, and a heatmap displayed the concordance and discordance of ranking orders across the whole cohort and sex-specific subgroups for both MASLD endpoints (i.e., MASLD presence and steatosis severity).

### Computational environment and code availability

All statistical analyses were performed on the overall dataset and separately for men and women in Python (version 3.11) using essential libraries such as scikit-learn (for modeling and evaluation), pandas and numpy (for data handling), and matplotlib and seaborn (for visualization). Algorithms training and performance assessments were conducted on a 13th Gen Intel Core i7—13620H CPU and 16 GB of RAM. The complete implementation scripts for model construction, threshold optimization, and performance evaluation are publicly accessible on GitHub: https://github.com/Mohamad-Jamalinia/AI-MASLD-Prediction.git.

## Results

### Population and sampling

A total of 446 participants (182 men and 264 women) were included, with a mean age of 52.9 years (51.9 years in men and 53.7 years in women). Among them, 284 participants had MASLD, of whom 184 participants had mild steatosis, and 100 participants had moderate-to-severe steatosis (as assessed by ultrasonography). Baseline characteristics of participants are summarized in Supplementary Tables 1–2. The dataset was randomly divided into training (n = 312, 70%) and testing subsets (n = 134, 30%), using stratified sampling to maintain the distribution of MASLD cases across both datasets. Sex-specific models were developed by splitting participants into men (n = 182) and women (n = 264), resulting in 127 men and 185 women in the training sets, and 55 men and 79 women in the testing sets, with MASLD prevalence proportionally preserved within each group.

### Model development and performance

**Fig. 1:**
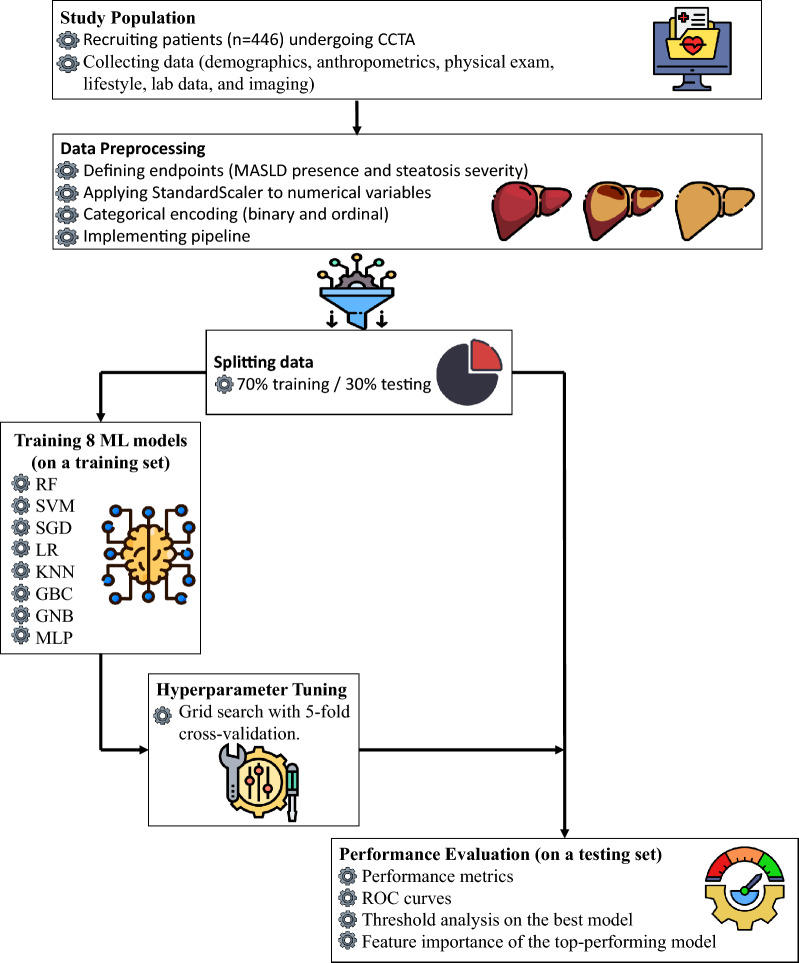
Study workflow for MASLD prediction using machine learning. The entire process was conducted for each of the two MASLD endpoints (MASLD presence and steatosis severity), both in the overall dataset and separately within the male and female subgroups. MASLD: Metabolic dysfunction-associated steatotic liver disease, CCTA: coronary computed tomography angiography, ML: machine learning, RF: Random Forest, SGD: Stochastic Gradient Descent Classifier, LR: Logistic Regression, KNN: K-Nearest Neighbors Classifier, GBC: Gradient Boosting Classifier, GNB: Gaussian Naive Bayes, MLP: Multi-Layer Perceptron Classifier, SVM: Support Vector Machine, ROC: receiver operating characteristic

**Fig. 2:**
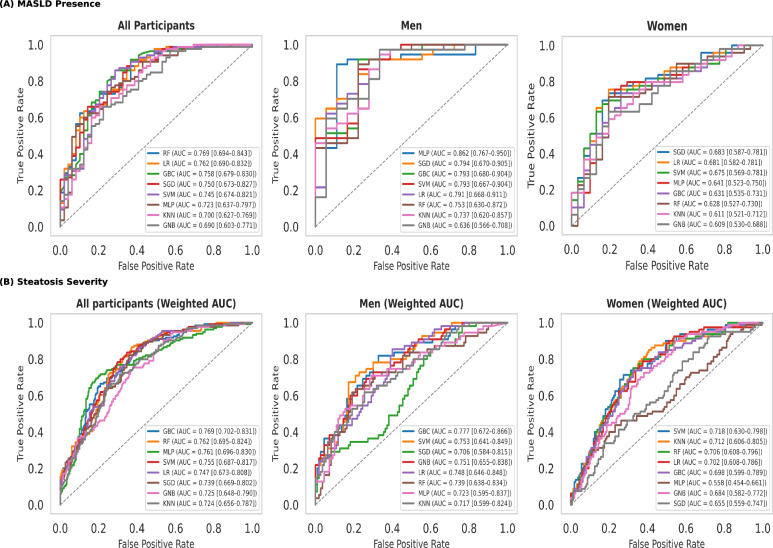
ROC curves demonstrate the performance of eight machine learning models in predicting MASLD presence and steatosis severity in the unseen test dataset. Each model was trained using optimal hyperparameters identified through a fivefold grid search. ROC curves are presented for the overall population and the male and female subgroups. MASLD: metabolic dysfunction-associated steatotic liver disease, RF: Random Forest, SGD: Stochastic Gradient Descent Classifier, LR: Logistic Regression, KNN: K-Nearest Neighbors Classifier, GBC: Gradient Boosting Classifier, GNB: Gaussian Naive Bayes, MLP: Multi-Layer Perceptron Classifier, SVM: Support Vector Machine, AUC: area under the receiver operating characteristic curve

Eight ML algorithms were trained on the development dataset. Hyperparameter tuning was performed using fivefold cross-validated grid search; the best-performing parameter combinations are shown in Supplementary Tables 3—4. Final performance assessment—including AUC, F1 score, sensitivity, specificity, PPV, and NPV with 95% confidence intervals—was conducted on the test set (Supplementary Tables 5—6). The complete training and evaluation pipeline is illustrated in Fig. 1. Among the eight models evaluated (Fig. 2), the top-performing classifiers for each outcome and subgroup are summarized in Table 1. For binary MASLD classification (no MASLD vs. MASLD presence), RF demonstrated the highest performance in the overall population (AUC = 0.769; F1 score = 0.856). Sex-specific modeling improved predictive accuracy in men, where GBC achieved the best performance (AUC = 0.793; F1 score = 0.897). In contrast, in women, performance metrics decreased, where LR performed best (AUC = 0.681; F1 score = 0.794). For multiclass classification of steatosis severity (no MASLD vs. MASLD with mild steatosis vs. MASLD with moderate to severe steatosis), MLP achieved the highest performance in the overall cohort (AUC = 0.761; F1 score = 0.671). Sex-specific models showed decreased performance in both sexes, with a difference more pronounced in women: MLP performed best in men (AUC = 0.723; F1 score = 0.608), whereas SVM was the top-performing model in women (AUC = 0.718; F1 score = 0.571). Our sensitivity analysis using the stratified fivefold cross-validation also confirmed the performance gap observed between men and women (Supplementary Table 7).

**Table 1:** Top-performing machine learning models for predicting MASLD presence and steatosis severity in the overall population, in men, and in women

Model(s)	AUC (95%CI)	F1 score (95%CI)	Sensitivity (95%CI)	Specificity (95%CI)	NPV (95%CI)	PPV (95%CI)
**Binary classification** (no MASLD / any degree MASLD)						
Overall (RF)	0.769 (0.694–0.843)	0.856 (0.798–0.905)	0.906 (0.840–0.963)	0.638 (0.500–0.772)	0.794 (0.667–0.917)	0.809 (0.736–0.885)
Men (GBC)	0.793 (0.680–0.904)	0.897 (0.815–0.964)	0.973 (0.912–0.999)	0.608 (0.375–0.846)	0.916 (0.714–0.999)	0.834 (0.714–0.932)
Women (LR)	0.681 (0.582–0.781)	0.794 (0.700–0.873)	0.877 (0.782–0.962)	0.482 (0.310–0.667)	0.710 (0.500–0.900)	0.728 (0.615–0.836)
**Multiclass classification** (no MASLD / mild MASLD / moderate to severe MASLD)						
Overall (MLP)	0.761 (0.696–0.830)	0.671 (0.597–0.750)	0.670 (0.597–0.746)	0.811 (0.756–0.862)	0.678 (0.602–0.755)	0.812 (0.756–0.862)
Men (MLP)	0.723 (0.595–0.837)	0.608 (0.468–0.742)	0.616 (0.491–0.745)	0.803 (0.724–0.874)	0.625 (0.482–0.760)	0.795 (0.697–0.873)
Women (SVM)	0.718 (0.630–0.798)	0.571 (0.448–0.685)	0.603 (0.487–0.713)	0.724 (0.644–0.796)	0.624 (0.458–0.753)	0.775 (0.691–0.856)

All metrics for multiclass classification are reported as weighted averages based on class support. MASLD severity was defined according to ultrasonographic grading of hepatic steatosis

MASLD: metabolic dysfunction-associated steatotic liver disease, RF: random forest, LR: logistic regression, MLP: multi-layer perceptron classifier, SVM: support vector machine, AUC: area under the receiver operating characteristic curve, CI: confidence interval, F1 score: harmonic mean of precision and recall, Sensitivity: true positive rate, Specificity: true negative rate, NPV: negative predictive value, PPV: positive predictive value

### Threshold-based interpretation

Threshold analyses were conducted for the top-performing algorithms in the binary classification task (no MASLD vs. MASLD presence) across the overall population and sex-specific subgroups (Table 2). Performance was evaluated at optimal, rule-in, and rule-out thresholds to assess the model’s utility for general classification, case confirmation, and the exclusion of MASLD, respectively.

**Table 2:** Threshold analysis of the top-performing machine learning model for binary classification of MASLD presence (no MASLD vs. any degree MASLD) in the overall population, in men, and in women

Group(s)	Threshold*	Sensitivity (95%CI)	Specificity (95%CI)	PPV (95% CI)	NPV (95% CI)	F1 score (95% CI)	Youden index (95%CI)
Overall (RF)							
Optimal cut-off	0.57	0.859 (0.784–0.930)	0.716 (0.596–0.830)	0.839 (0.759–0.907)	0.747 (0.620–0.867)	0.848 (0.792–0.903)	0.575 (0.438–0.715)
Rule-in cut-off	0.90	0.247 (0.157–0.345)	0.980 (0.936–0.999)	0.956 (0.850–0.999)	0.429 (0.343–0.514)	0.390 (0.268–0.508)	0.227 (0.130–0.333)
Rule-out cut-off	0.25	0.988 (0.962–0.999)	0.328 (0.192–0.457)	0.719 (0.638–0.798)	0.941 (0.800–0.999)	0.832 (0.775–0.884)	0.316 (0.180–0.445)
Men (GBC)							
Optimal cut-off	0.67	0.760 (0.615–0.892)	0.943 (0.812–0.999)	0.965 (0.886–0.999)	0.657 (0.458–0.833)	0.848 (0.746–0.935)	0.703 (0.516–0.865)
Rule-in cut-off	0.67	0.760 (0.615–0.892)	0.943 (0.812–0.999)	0.965 (0.886–0.999)	0.657 (0.458–0.833)	0.848 (0.746–0.935)	0.703 (0.516–0.865)
Rule-out cut-off	0.53	0.973 (0.906–0.999)	0.718 (0.500–0.909)	0.876 (0.769–0.974)	0.928 (0.750–0.999)	0.921 (0.853–0.976)	0.691 (0.446–0.894)
Women (LR)							
Optimal cut-off	0.49	0.753 (0.633–0.872)	0.807 (0.675–0.938)	0.860 (0.756–0.954)	0.676 (0.513–0.833)	0.801 (0.706–0.884)	0.561 (0.371–0.738)
Rule-in cut-off	0.64	0.364 (0.229–0.510)	0.934 (0.840–0.999)	0.896 (0.737–0.999)	0.484 (0.362–0.615)	0.514 (0.361–0.658)	0.298 (0.133–0.454)
Rule-out cut-off	0.21	0.980 (0.935–0.999)	0.192 (0.059–0.345)	0.659 (0.553–0.765)	0.853 (0.500–0.999)	0.786 (0.707–0.859)	0.172 (0.033–0.323)

^*^Classification thresholds were evaluated by normalizing decision scores to [0,1] and calculating sensitivity, specificity, PPV, NPV, and F1 score at 0.01 intervals. The optimal threshold was assigned to the highest Youden’s index, whereas the “rule-in” and “rule-out” thresholds were based on clinically relevant criteria as follows: rule-in was defined as the threshold with the highest PPV among participants with specificity > 90%, whereas rule-out was defined as the highest NPV among those with sensitivity > 90%. In men, the optimal threshold coincided with the rule-in threshold, resulting in identical performance estimates

MASLD: metabolic dysfunction-associated steatotic liver disease, RF: random forest, GBC: gradient boosting classifier, LR: logistic regression, PPV: positive predictive value, NPV: negative predictive value

At the optimal threshold, the ML models demonstrated balanced performance across the overall population and by sex. In the overall population, sensitivity was high (0.859) and specificity moderate (0.716), with strong PPV (0.839) and NPV (0.747), thus reflecting reliable detection of MASLD and reasonable exclusion of non-MASLD cases. In men, the optimal threshold showed slightly lower sensitivity (0.760) but very high specificity (0.943), resulting in excellent PPV (0.965) but lower NPV (0.657), indicating strong performance in confirming the presence of MASLD. In women, sensitivity (0.753) and specificity (0.807) were balanced, with PPV 0.860 and NPV 0.676, suggesting good overall predictive accuracy, though lower than in men for specificity.

At rule-in thresholds, in the overall population, specificity reached 0.980 and PPV 0.956, while sensitivity dropped to 0.247, showing that many MASLD cases would be missed, but positive predictions are highly reliable. In men, the rule-in threshold was equal to the optimal threshold, maintaining specificity 0.943 and PPV 0.965, demonstrating strong confirmatory performance. In women, specificity increased to 0.934 and PPV to 0.896, but sensitivity decreased to 0.364, indicating that the model identifies MASLD cases with high confidence but misses a substantial portion of affected women.

At the rule-out thresholds, in the overall population, sensitivity was 0.988 and NPV 0.941, though specificity was low (0.328), reflecting many false positives. In men, sensitivity increased to 0.973 and NPV to 0.928, with acceptable specificity (0.718), indicating a strong ability to exclude MASLD. In women, sensitivity was similarly high (0.980) and NPV moderate (0.853), but specificity was very low (0.192), suggesting that while MASLD can be confidently ruled out, many women without MASLD may be incorrectly classified as positive.

### Feature importance analysis

**Fig. 3:**
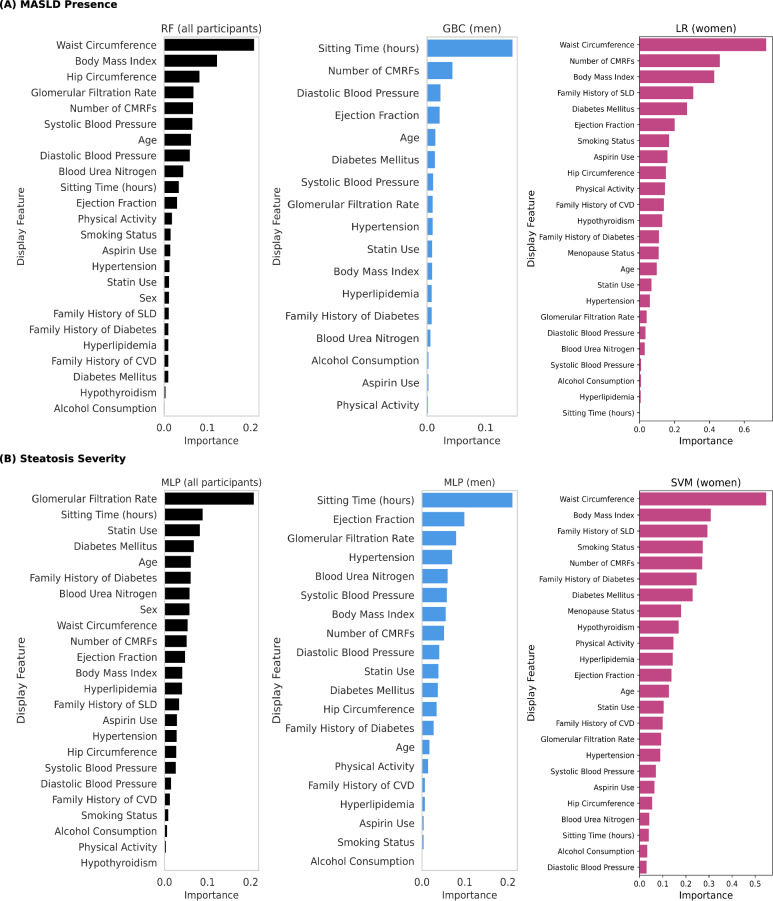
Feature importance plots for the best-performing machine learning models for predicting MASLD presence and steatosis severity in the overall population and sex-specific subgroups. For each subset, variables are ranked according to model-derived importance scores, with higher values indicating greater contribution to model prediction. Feature-importance units differ by algorithm: absolute model coefficients for support vector machine (SVM) and logistic regression (LR); impurity-based importance for random forest (RF) and gradient boosting classifier (GBC); and permutation importance for multilayer perceptron (MLP). These importance measures reflect the magnitude of a variable’s contribution to prediction and do not indicate directionality (i.e., whether the association is positive or negative). Postmenopausal status was defined using an age-based proxy (≥ 50 years). CMRF: cardiometabolic risk factors, MASLD: metabolic dysfunction-associated steatotic liver disease, CVD: cardiovascular disease

**Fig. 4:**
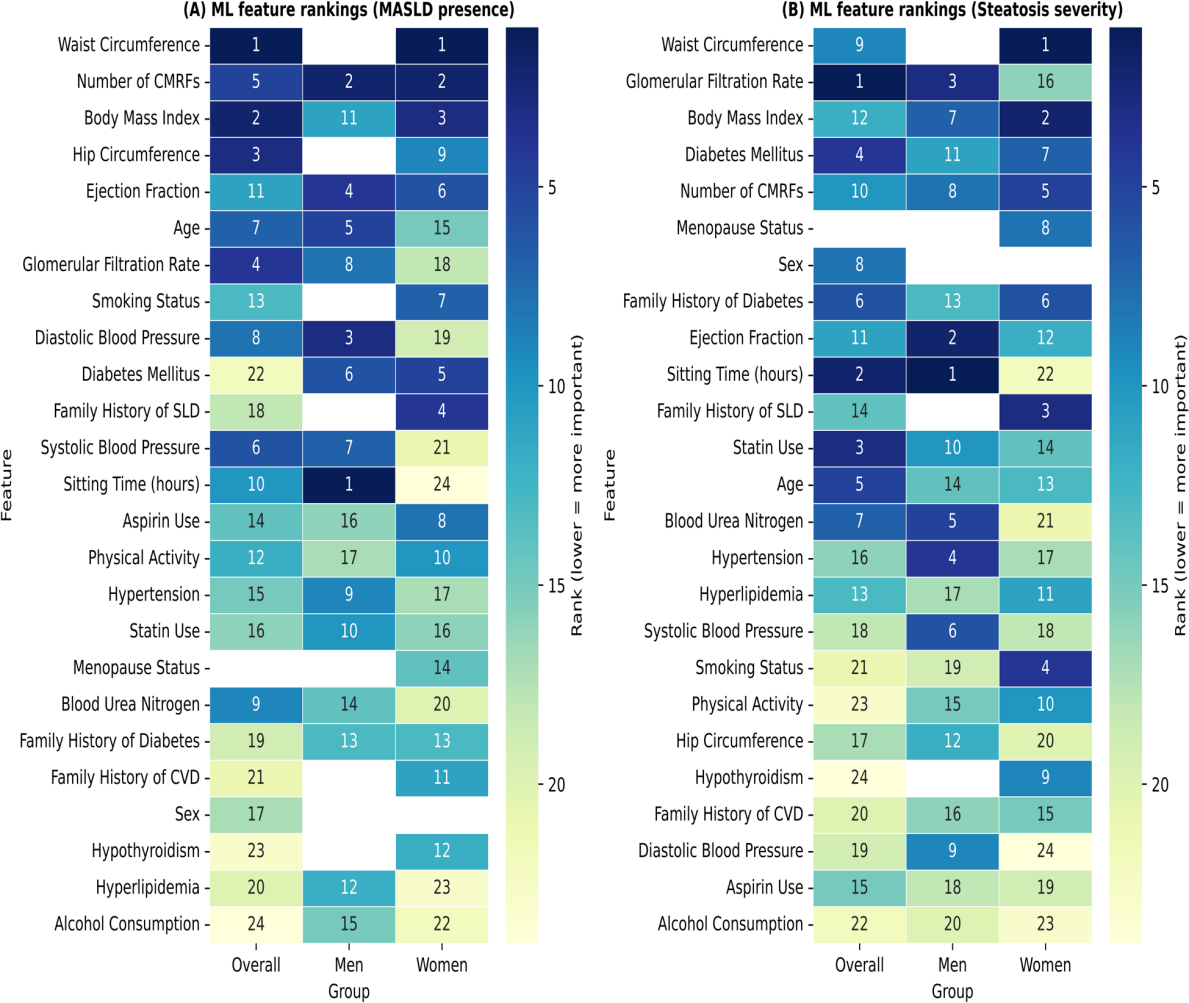
Heatmap illustrating discordance in machine-learning–derived feature importance rankings across overall and sex-specific subgroups for the two MASLD-related endpoints (MASLD presence and steatosis severity). Color intensity represents relative feature importance, with darker shading indicating higher importance. Numeric values correspond to the rank order of each feature, with lower values indicating higher importance and higher values indicating lower importance, consistent with the rankings shown in Fig. 3. These rankings reflect relative contribution to model prediction rather than direction of association. Postmenopausal status was defined using an age-based proxy (≥ 50 years). CMRF: cardiometabolic risk factors, MASLD: metabolic dysfunction-associated steatotic liver disease, CVD: cardiovascular disease

Feature importance analysis identified distinct sets of predictors for MASLD presence and steatosis severity between the sexes (Figs. 3, 4).

In the overall population, the number of cardiometabolic risk factors (CMRFs) ranked among the top predictors of MASLD presence and steatosis severity. Blood pressure and age contributed meaningfully, alongside T2DM, hypertension, and dyslipidemia. In men, the number of CMRFs and diastolic blood pressure were among the most influential risk factors, followed by systolic blood pressure, T2DM, and age. In women, CMRFs, T2DM, and dyslipidemia remained strongly predictive. Age-based menopause proxy ranked higher than age in women, thus reflecting the greater impact of the hormonal transition on metabolic and hepatic function. Hypothyroidism showed mid-range importance in women, particularly in the presence model, while in men, its predictive importance was minimal.

Anthropometric parameters dominated the overall MASLD presence model, with BMI, waist circumference, and hip circumference showing the highest importance. In women, waist circumference and BMI were the most discriminative features. In men, measures of regional adiposity, such as waist and hip circumference, had lower relative importance and were automatically excluded from some final algorithms, while behavioral variables and renal function parameters played a more prominent role in MASLD prediction. Behavioral variables showed sex-dependent contributions. Sitting time was the most influential predictor among men, ranking first in both presence and severity models. In women, physical activity and sitting time ranked lower, suggesting more modest effects than adiposity and familial factors.

Renal function parameters emerged as important determinants, particularly for steatosis severity. eGFR ranked among the highest features overall, followed by BUN. In men, eGFR and BUN were strongly associated with both presence and severity prediction, whereas in women, renal markers were less influential, ranking below adiposity and familial factors.

Positive family histories for steatotic liver diseases (SLD), T2DM, and CVDs contributed moderately to MASLD prediction. In women, family history for SLD was among the most relevant predictors, reflecting potential shared metabolic or genetic predisposition. In men, family history for T2DM ranked moderately, while family history for CVDs appeared less influential overall.

Left ventricular ejection fraction (LVEF) on echocardiography contributed to both the MASLD presence and steatosis severity predictions but was more important in men. LVEF ranked among the top features in male-severity models, suggesting potential cardiac–metabolic interactions that may differ by sex. In contrast, LVEF had a moderate-to-low influence on female models.

Aspirin and statin use contributed moderately in all predictive models, ranking higher in steatosis severity prediction than in MASLD presence models. Statin use was more influential in men, while both medications showed comparable mid-level importance in women. Smoking history displayed moderate importance overall and ranked higher in women, where it contributed more meaningfully to steatosis severity prediction. Alcohol consumption consistently ranked among the lowest predictors in all prediction models, showing minimal discriminative value.

Collectively, these findings strongly suggest that MASLD predictors are sex-specific and domain-dependent. Abdominal adiposity and cardiometabolic burden are universal drivers of disease risk, yet their interactions differ between men and women. Male-specific models emphasize behavioral and cardiorenal features, whereas female-specific models are dominated by adiposity, hormonal, and familial–metabolic factors.

## Discussion

### Main findings

In the present study, we developed for the first time eight sex-specific ML models for risk prediction of MASLD using clinical, metabolic, and behavioral variables obtained from standard clinical practice in a medically referred cardiology population. We found that men and women exhibited distinct learning patterns, reflected by marked differences in feature importance rankings across algorithms. In men, model performance for predicting MASLD relied predominantly on behavioral factors and CKM variables, whereas in women, the algorithms drew more heavily on adiposity measures, metabolic traits, and familial characteristics. These divergent patterns underscore the fundamentally different biological and environmental drivers of MASLD between men and women. Furthermore, sex-specific training improved discrimination in men, and only the male models achieved clinically meaningful thresholds for both rule-in and rule-out applications. By contrast, discrimination decreased in the female cohort, and none of the women-specific models reached performance levels suitable for clinical use. A similar learning pattern emerged when evaluating the severity of steatosis; however, sex-stratified modeling did not improve discrimination of steatosis severity in either group.

We believe that the suboptimal performance of these models in women likely reflects long-standing gaps in how risk factors for MASLD are captured in routine clinical practice and research datasets [25]. Women have unique hormonal, metabolic, and life-course-related determinants of MASLD that are often underrepresented or inadequately measured, thereby limiting the learning capacity of algorithms [25]. Overall, our findings emphasize the urgent need for sex-stratified data processing and modeling for complex, multidimensional diseases, such as MASLD.

### AI and sex-specific learning patterns

The sex-specific learning patterns revealed in each model align with the growing recognition that MASLD has distinct pathobiological and metabolic trajectories in men and women [26]. For men, renal function parameters, especially eGFR, were consistently among the highest-ranked features during model training. This finding is consistent with emerging evidence that men are exposed to substantially higher rates of MASLD-related CKD [13]. For women, instead, predictive algorithms placed greater weight on family and life-history variables. A family history for SLD was among the highest-ranked predictors among female algorithms, likely due to several factors. Beyond classical genetic predisposition, familial patterns may partially capture heritable reproductive traits (e.g., age at menarche and at menopausal transition, and duration of estrogen exposure) that have been strongly associated with MASLD susceptibility [27]. Shared lifestyles and environmental factors may also influence the family’s influence on health. Therefore, the impact of family history on female-specific models is likely a combination of genetic, hormonal, and environmental pathways that shape the risk of MASLD across a woman’s lifetime.

We also found distinct differences in the contribution of physical inactivity. While sedentary behavior is a known risk factor for MASLD [28], physical inactivity indices were ranked among the most informative predictors in men. In contrast, the female algorithms relied more heavily on adiposity markers, such as waist circumference and BMI. Most importantly, these learning patterns were not evident in models that included sex as the sole predictor. As shown in Fig. 4, sex-stratified training revealed non-linear interactions and risk structures that would typically be masked in pooled analyses. This further supports the limitation of using sex as a simple binary covariate and provides methodological value to generate sex-specific models [29].

### Clinical implications

The sex-specific learning patterns observed in our study may have important clinical implications for improving the detection of MASLD. Training separate models for men and women enabled calibration to sex-specific clinical and metabolic distributions, resulting in good performance in men but suboptimal discrimination in women. Our findings align with prior work showing that ML classifiers for liver disease can generate false-negative rates up to 24% higher in women [30]. A possible explanation for this disparity is that most models, when stratified, do not account for female-specific risk factors unique to MASLD, including reproductive history (e.g., parity, number of pregnancies, gestational diabetes mellitus, lactation, and its duration) and hormonal transitions across the lifespan [27]. These biological factors, while strongly associated with the development and progression of MASLD [27], are rarely available in health records or prediction frameworks, which may help explain the lower algorithmic performance in women.

Similar limitations can also exist with widely used non-invasive biomarkers [31]. Blood-based scores such as FLI, FIB-4, and NFS (NAFLD Fibrosis Score) use unisex cut-offs despite physiological sex differences [15]. There is evidence that the best thresholds for FLI are lower in women [16]. FIB-4 index, on the one hand, may overestimate the risk in premenopausal women, but may underestimate it in postmenopausal women [32]. Given that MASLD-related complications (liver decompensation, HCC, CVD, CKD) may differ by sex as well, using a unisex threshold can lead to under- and over-classified risk, which can have meaningful downstream implications for the process of clinical decision-making. From a clinical perspective, this implies that current MASLD risk calculators could carry a high risk of misclassifying women, and that results in women with MASLD should be interpreted with added caution [33]. This is especially notable given the growing global burden of MASLD (~ 1.3 billion adults) and its increasing contribution to liver-related and extrahepatic morbidity and mortality [34]. Collectively, therefore, the results of our study support the importance of incorporating sex-stratified data processing into future AI-based MASLD prediction models to ultimately reduce diagnostic inequities and improve screening and risk assessment accuracy.

### Strengths and limitations

This proof-of-concept study has important strengths. To our knowledge, it is the first study to develop and evaluate sex-specific ML algorithms for predicting MASLD presence and steatosis severity. As a proof-of-concept analysis, it shows that sex-stratified data processing may enhance the detection of a complex, multidimensional condition such as MASLD in men, while also revealing the limitations of traditional datasets for accurately detecting MASLD in women. Our insights provide a valuable foundation for future work aimed at improving diagnostic equity within a population (women) that has been historically underdiagnosed in pooled risk estimates [29, 33]. We also evaluated a large range of ML architectures and performed careful hyperparameter tuning using grid search with cross-validation. Our final models were assessed on unseen data, increasing the robustness and reliability of the findings. By predicting MASLD presence and steatosis severity, we also characterized sex-specific learning patterns across multiple disease dimensions. Finally, the consistency of results across stratified fivefold cross-validation gave more credence to the performance gap observed between men and women.

Despite these strengths, several limitations should be acknowledged. This was a single-center study of patients referred for CCTA, representing a higher-risk, cardiology-focused population with an increased prevalence of metabolic dysfunction. The observed MASLD prevalence in this cohort (~ two in three) is approximately double than that reported in population-based estimates (~ one in three), reflecting both referral bias and the selection of patients with established cardiovascular risk. Consequently, the findings may not generalize to community- or primary-care populations. Until broader external validation with population-based cohorts is performed, the applicability of the sex-specific ML models should be considered primarily within populations with similar cardiometabolic risk profiles. The sample sizes within sex-specific subgroups were relatively modest, and the combination of limited sample sizes and class imbalance could have affected the stability of algorithm outputs. Although we applied stratified sampling, bootstrap resampling, and cross-validation to mitigate these effects, reduced algorithm performance in some subgroups, particularly among women, may be partly explained by these factors. Our analysis was also constrained by the variables available in the clinical database. Established metabolic risk parameters, such as homeostatic model assessment for insulin resistance (HOMA-IR), were not available, and several female-specific variables, including parity, age at menarche, lactation history, oral contraceptive use, hormone replacement therapy, and detailed hormonal profiling, were not collected. In men, androgen-related measures, such as testosterone levels, were also not measured [27]. The absence of these sex-specific variables could have biased model predictions, particularly in women, potentially contributing to observed sex differences in algorithm performance. Menopausal status was approximated using an age threshold, potentially misclassifying some women. According to our definition, the prevalence of premenopausal women in our cohort was relatively low (~ 32%), limiting the algorithm’s ability to fully capture premenopause-specific risk patterns. Incorporating richer phenotyping and more comprehensive hormonal and metabolic profiling in future studies, particularly those focused on premenopausal women, will be important to strengthen sex- and menopause-specific predictive models. Interpretation of ML performance metrics and feature importance also warrants caution. Analyses were cross-sectional, and high feature importance does not imply biological causality; rather, it reflects the variable’s discriminative contribution within the dataset’s structure and its interactions with other predictors. Finally, our definition of MASLD severity was based on ultrasonographic steatosis extent rather than fibrosis stage or biopsy-confirmed steatohepatitis. While ultrasound is widely used clinically, it has recognized limitations, including low sensitivity in individuals with high BMI and a non-linear relationship with liver fibrosis severity, which is the strongest predictor of hepatic and extrahepatic outcomes [35]. In advanced MASLD, hepatic steatosis may paradoxically decrease while accumulation of toxic lipids continues, meaning that steatosis extent may not reliably reflect fibrosis risk [36]; therefore, we would expect that patients with clinically significant liver fibrosis could have fallen into either the mild or moderate–severe steatosis groups, thereby introducing the possibility for severity misclassification and limiting the ability of ML models to identify sex-specific severity patterns. To address these limitations, future studies should plan to incorporate longitudinal follow-up data with clinically meaningful endpoints of MASLD, particularly liver fibrosis progression, to assess whether sex-specific ML models improve prognostic accuracy and reclassification metrics over time.

## Conclusion

The findings of our study provide novel evidence of sex-specific learning patterns in predicting MASLD presence and steatosis severity, with distinct performance outcomes. Overall, sex-stratified model training improved predictive accuracy for men, while prediction performance remained suboptimal for women. Our results suggest that one-size-fits-all models fail to account for sex-specific pathobiology of MASLD and likely are underestimating risk factors for women, reinforcing previously established inequities in medical practice from the pooled, sex-agnostic data. Specifically, biological, hormonal, and reproductive risk factors in women have historically been excluded from traditional datasets used for model development [25], with implications for existing risk calculators and ML frameworks that may systematically misclassify female patients. Based on these findings, the present study supports adopting sex-stratified data processing in MASLD risk prediction and suggests (re)evaluation of non-invasive risk scores for diagnostic accuracy in sex-specific versus pooled sex-agnostic diagnosis. Notably, the addition of longitudinal follow-up, which can be feasibly focused on liver fibrosis progression, and the inclusion of previously excluded female-specific MASLD risk factors, such as reproductive history and hormonal transition, will improve algorithm performance in women and minimize sex-based diagnostic inequities in current MASLD algorithms.

## Supplementary Information

Supplementary Material 1

## Data Availability

The datasets used and/or analysed during the current study are available from the corresponding author on reasonable request.
