# Supplementary material for: Artificial intelligence predicts sex-specific risk of metabolic dysfunction-associated steatotic liver disease
Source: Biol Sex Differ. 2026 May 8;17:126. doi: 10.1186/s13293-026-00917-6 (PMC13321611; doi:10.1186/s13293-026-00917-6)
Supplement: Supplementary file 1 — Supplementary Material 1 [file 13293_2026_917_MOESM1_ESM.docx]

**Supplementary Materials**

**Supplementary Table 1**. Baseline characteristics in sex-specific subgroups.

**Supplementary Table 2.** Baseline characteristics according to steatosis severity.

**Supplementary Table 3.** Optimal hyperparameters of machine learning models for binary classification of MASLD presence (no MASLD vs. any degree MASLD) in the overall population and sex-specific subgroups.

**Supplementary Table 4.** Optimal hyperparameters of machine learning models for multiclass classification of steatosis severity (no MASLD vs. mild MASLD vs. moderate to severe MASLD) in the overall population and sex-specific subgroups.

**Supplementary Table 5.** Performance metrics of machine learning models for binary classification of MASLD presence (no MASLD vs. any degree MASLD) on the test set in the overall population and sex-specific subgroups.

**Supplementary Table 6.** Weighted performance metrics of machine learning models for multiclass classification of steatosis severity (no MASLD vs. mild MASLD vs. moderate to severe MASLD) on the test set in the overall population and sex-specific subgroups.

**Supplementary Table 7.** Sensitivity analysis for top-performing machine learning models using stratified 5-fold cross-validation for predicting MASLD presence and steatosis severity in the overall population, in men, and in women.

| **Supplementary Table 1**. **Baseline characteristics in sex-specific subgroups.** | | | |
| --- | --- | --- | --- |
|  | **Female**  **(N = 264)** | **Male**  **(N = 182)** | **p-value** |
| Age (years) | 53.71 ± 9.26 | 51.9 ± 10.78 | 0.058 |
| Weight (kg) | 71.42 ± 11.66 | 79.42 ± 12.76 | 0.001 |
| Height (cm) | 158.14 ± 6.24 | 172.6 ± 8.21 | 0.001 |
| Hip circumference (cm) | 102.93 ± 12.05 | 98.7 ± 10.79 | 0.001 |
| Cardiometabolic risk factors (CMRF) |  |  |  |
| Diabetes (Yes/No) % | 53 (20.1%) / 211 (79.9%) | 16 (8.8%) / 166 (91.2%) | 0.002 |
| Dyslipidemia (Yes/No) % | 59 (22.3%) / 205 (77.7%) | 28 (15.4%) / 154 (84.6%) | 0.089 |
| Hypertension (Yes/No) % | 120 (45.5%) / 144 (54.5%) | 58 (31.9%) / 124 (68.1%) | 0.005 |
| Systolic blood pressure (mm Hg) | 138.80 ± 19.40 | 137.05 ± 12.95 | 0.287 |
| Diastolic blood pressure (mm Hg) | 85.29 ± 11.88 | 86.22 ± 9.87 | 0.386 |
| Body mass index (kg/m^2^) | 28.58 ± 4.53 | 26.66 ± 3.95 | 0.001 |
| Waist circumference (cm) | 90.21 ± 11.78 | 91.31 ± 10.71 | 0.313 |
| Number of CMRF | 1.76 ± 1.01 | 1.28 ± 0.95 | 0.001 |
| Cardiac function |  |  |  |
| Ejection fraction % | 56.04 ± 3.79 | 55.0 ± 5.01 | 0.013 |
| Renal function |  |  |  |
| Glomerular filtration rate (ml/min) | 83.68 ± 25.0 | 93.6 ± 24.63 | 0.001 |
| Blood urea nitrogen (mg/dl) | 13.55 ± 4.13 | 15.05 ± 4.59 | 0.001 |
| Liver parameter |  |  |  |
| MASLD steatosis grading (0/1/2-3) % | 102 (38.6%) / 113 (42.8%) / 49(18.6%) | 60 (33.0%) / 71 (39.0%) / 51(28.0%) | 0.060 |
| Female specific variable |  |  |  |
| Menopause (age-based proxy ≥50 years) (Yes/No) % | 180 (68.2%) / 84 (31.8%) | — | — |
| Family history |  |  |  |
| Cardiovascular disease (Yes/No) % | 115 (43.6%) / 149 (56.4%) | 70 (38.5%) / 112 (61.5%) | 0.329 |
| Diabetes (Yes/No) % | 84 (31.8%) / 180 (68.2%) | 43 (23.6%) / 139 (76.4%) | 0.076 |
| Steatotic liver disease (Yes/No) % | 40 (15.2%) / 224 (84.8%) | 6 (3.3%) / 176 (96.7%) | 0.001 |
| Sedentary behavior |  |  |  |
| Physical activity (Low/Mild/Moderate/Severe) % | 156 (59.1%) / 77 (29.2%) / 9(3.4%) / 22(8.3%) | 93 (51.1%) / 41 (22.5%) / 15(8.2%) / 33(18.1%) | 0.001 |
| Sitting time (hours) | 5.95 ± 2.3 | 6.64 ± 2.97 | 0.006 |
| Medications |  |  |  |
| Statin (Yes/No) % | 114 (43.2%) / 150 (56.8%) | 54 (29.7%) / 128 (70.3%) | 0.005 |
| Aspirin (Yes/No) % | 86 (32.6%) / 178 (67.4%) | 47 (25.8%) / 135 (74.2%) | 0.154 |
| Levothyroxine (Yes/No) % | 32 (12.1%) / 232 (87.9%) | 8 (4.4%) / 174 (95.6%) | 0.008 |
| Substance abuse |  |  |  |
| Smoking (Yes/No) % | 47 (17.8%) / 217 (82.2%) | 70 (38.5%) / 112 (61.5%) | 0.001 |
| Alcohol (Yes/No) % | 2 (0.8%) / 262 (99.2%) | 26 (14.3%) / 156 (85.7%) | 0.001 |
| *Abbreviations*: CKD: chronic kidney disease, MASLD: Metabolic dysfunction-associated steatotic liver disease. | | | |

| **Supplementary Table 2. Baseline characteristics according to steatosis severity.** | | | | |
| --- | --- | --- | --- | --- |
|  | **No MASLD**  **(N = 162)** | **Mild MASLD**  **(N = 184)** | **Moderate to severe MASLD (N = 100)** | **p-value** |
| Age (years) | 51.33 ± 10.48^a^ | 53.59 ± 9.31^b^ | 54.49 ± 9.88^c^ | 0.024 |
| Sex (Male / Female) % | 60 (37.0%) / 102 (63.0%) | 71 (39.0%) / 113 (61.0%) | 51 (51.0%) / 49 (49.0%) | 0.060 |
| Weight (kg) | 67.17 ± 11.02^a^ | 76.99 ± 10.53^b^ | 82.6 ± 12.66^c^ | 0.001 |
| Height (cm) | 163.18 ± 9.59 | 164.27 ± 10.22 | 165.01 ± 10.46 | 0.332 |
| Hip circumference (HC) (cm) | 95.4 ± 11.51^a^ | 103.39 ± 10.47^b^ | 106.61 ± 10.32^c^ | 0.001 |
| Cardiometabolic risk factors (CMRF) |  |  |  |  |
| Diabetes (Yes/No) % | 12 (7.0%) / 150 (93.0%) | 29 (16.0%) / 155 (84.0%) | 28 (28.0%) / 72 (72.0%) | 0.001 |
| Dyslipidemia (Yes/No) % | 17 (10.0%) / 145 (90.0%) | 40 (22.0%) / 144 (78.0%) | 30 (30.0%) / 70 (70.0%) | 0.001 |
| Hypertension (Yes/No) % | 47 (28.9%) / 115 (71.1%) | 78 (42.1%) / 106 (57.9%) | 53 (53.0%) / 47 (47.0%) | 0.001 |
| Systolic blood pressure (SBP) (mm Hg) | 134.80 ± 16.42^a^ | 138.90 ± 17.20^b^ | 141.92 ± 17.05^c^ | 0.003 |
| Diastolic blood pressure (DBP) (mm Hg) | 83.25 ± 10.16^a^ | 86.36 ± 11.24^b^ | 88.33 ± 11.63^c^ |  |
| Body mass index (BMI) (kg/m^2^) | 25.26 ± 3.81^a^ | 28.61 ± 3.72^b^ | 30.42 ± 4.38^c^ | 0.001 |
| Waist circumference (WC) (cm) | 82.28 ± 9.08^a^ | 93.64 ± 9.48^b^ | 98.74 ± 9.07^c^ | 0.001 |
| Number of CMRF | 1.08 ± 0.87^a^ | 1.71 ± 0.93^b^ | 2.08 ± 1.03^c^ | 0.001 |
| Cardiac function |  |  |  |  |
| Ejection fraction % | 55.34 ± 4.63 | 56.03 ± 4.31 | 55.3 ± 3.95 | 0.245 |
| Renal function |  |  |  |  |
| Glomerular filtration rate (GFR ) (ml/min) | 83.66 ± 21.68^a^ | 88.73 ± 26.44^b^ | 92.49 ± 27.72^c^ | 0.018 |
| CKD staging (0-1/2/3) % | 58 (35.8%) / 86(53.1%) / 18(11.1%) | 80(43.5% / 85(46.2%) / 19(10.3%) | 50 (50.0%) / 37(37.0%) / 13(13.0%) | 0.139 |
| Blood urea nitrogen (mg/dl) | 14.24 ± 4.13 | 14.09 ± 4.3 | 14.18 ± 4.93 | 0.951 |
| Female specific variable |  |  |  |  |
| Menopause (age-based proxy ≥50 years)(Yes/No) % | 65 (63.7%) / 37 (36.3%) | 77 (68.1%) / 36 (31.9%) | 38 (77.6%) / 11 (22.4%) | 0.233 |
| Family history |  |  |  |  |
| Cardiovascular disease (Yes/No) % | 59 (36.0%) / 103 (64.0%) | 84 (46.0%) / 100 (54.0%) | 42 (42.0%) / 58 (58%) | 0.219 |
| Diabetes (Yes/No) % | 33 (20.0%) / 129 (80.0%) | 55 (30.0%) / 129 (70.0%) | 39 (39.0%) / 61 (61.0%) | 0.004 |
| Steatotic liver disease (Yes/No) % | 8 (5.0%) / 154 (95.0%) | 27 (15.0%) / 157 (85.0%) | 11 (11.0%) / 89 (89.0%) | 0.012 |
| Sedentary behavior |  |  |  |  |
| Physical activity (Low/Mild/Moderate/Severe)% | 90 (55.6%) / 37 (22.8%) / 7(4.3%) / 28(17.3%) | 104 (56.5%) / 60 (32.6%) / 8(4.3%) / 12(6.5%) | 55 (55.0%) / 21 (21.0%) / 9(9.0%) / 15(15.0%) | 0.011 |
| Sitting time (hours) | 349.94 ± 141.22 | 392.66 ± 166.26 | 378.4 ± 159.25 | 0.038 |
| Medications |  |  |  |  |
| Statin (Yes/No) % | 54 (33.0%) / 108 (67.0%) | 74 (40.0%) / 110 (60.0%) | 40 (40.0%) / 60 (60.0%) | 0.361 |
| Aspirin (Yes/No) % | 46 (28.0%) / 116 (72.0%) | 56 (30.0%) / 128 (70.0%) | 31 (31.0%) / 69 (69.0%) | 0.879 |
| Levothyroxine (Yes/No) % | 11 (7.0%) / 151 (93.0%) | 18 (10.0%) / 166 (90.0%) | 11 (11.0%) / 89 (89.0%) | 0.450 |
| Substance abuse |  |  |  |  |
| Smoking (Yes/No) % | 29 (18.0%) / 133 (82.0%) | 49 (27.0%) / 135 (73.0%) | 39 (39.0%) / 61 (61.0%) | 0.001 |
| Alcohol (Yes/No) % | 7 (4.0%) / 155 (96.0%) | 14 (8.0%) / 170 (92.0%) | 7 (7.0%) / 93 (93.0%) | 0.428 |
| *Abbreviations:* CKD: chronic kidney disease, MASLD: Metabolic dysfunction-associated steatotic liver disease.  ***Post-hoc analysis identifying specific subgroup differences in one-way ANOVA test:*** age (a vs. c), weight (a vs. b vs. c), HC (a vs. b vs. c), SBP (a vs. c), DBP (a vs. b vs. c), BMI (a vs. b vs. c), WC (a vs. b vs. c), number of CMRF (a vs. b vs. c), and eGFR (a vs. c). | | | | |

| **Supplementary Table 3. Optimal hyperparameters of machine learning models for binary classification of MASLD presence (no MASLD vs. any degree MASLD) in the overall population and sex-specific subgroups.** | | |
| --- | --- | --- |
| **ML algorithms** | **Optimal Hyperparameter** | |
| Overall | |  |
| Random Forest | max_depth=None, min_samples_split=2, n_estimators=200 | |
| SGD Classifier | alpha=0.01, eta0=0.1, learning_rate='adaptive', loss='hinge', penalty='l1' | |
| Logistic Regression | C=0.1, penalty='l2', solver='lbfgs' | |
| KNeighbors Classifier | leaf_size=20, n_neighbors=50, p=2, weights='distance' | |
| Gradient Boosting | learning_rate=0.05, max_depth=3, max_features='sqrt', n_estimators=100, subsample=0.8 | |
| GaussianNB | var_smoothing=1e-09 | |
| MLP Classifier | activation='tanh', alpha=0.0001, hidden_layer_sizes=(50, 50, 50), solver='adam' | |
| Support Vector Machine | C=0.1, gamma='scale', kernel='linear' | |
|  |  | |
| Men |  | |
| Random Forest | max_depth=None, min_samples_split=5, n_estimators=200 | |
| SGD Classifier | alpha=0.01, eta0=0.01, learning_rate='constant', loss='log_loss', penalty='l1' | |
| Logistic Regression | C=0.1, penalty='l2', solver='liblinear' | |
| KNeighbors Classifier | leaf_size=20, n_neighbors=50, p=1, weights='distance' | |
| Gradient Boosting | learning_rate=0.01, max_depth=3, max_features='sqrt', n_estimators=100, subsample=1.0 | |
| GaussianNB | var_smoothing=1e-09 | |
| MLP Classifier | activation='tanh', alpha=0.001, hidden_layer_sizes=(50,), solver='lbfgs' | |
| Support Vector Machine | C=0.1, gamma='auto', kernel='rbf' | |
|  |  | |
| Women |  | |
| Random Forest | max_depth=None, min_samples_split=5, n_estimators=100 | |
| SGD Classifier | alpha=0.01, eta0=0.01, learning_rate='adaptive', loss='log_loss', penalty='l1' | |
| Logistic Regression | C=0.1, penalty='l2', solver='lbfgs' | |
| KNeighbors Classifier | leaf_size=20, n_neighbors=50, p=1, weights='distance' | |
| Gradient Boosting | learning_rate=0.01, max_depth=3, max_features='sqrt', n_estimators=200, subsample=0.8 | |
| GaussianNB | var_smoothing=1e-09 | |
| MLP Classifier | activation='tanh', alpha=0.0001, hidden_layer_sizes=(50, 50), solver='adam' | |
| Support Vector Machine | C=0.1, gamma='auto', kernel='poly' | |
| ***Hyperparameter explanation:*** max_depth: Maximum depth of the tree; min_samples_split: Minimum number of samples required to split an internal node; n_estimators: Number of trees in the forest or boosting stages; alpha: Regularization strength for SGD and MLP classifiers; eta0: Initial learning rate for SGD; learning_rate: Learning rate schedule for weight updates (SGD); loss: Loss function used by the classifier; penalty: Regularization method; C: Inverse of regularization strength for Logistic Regression and SVM; solver: Algorithm to use in optimization; leaf_size: Leaf size parameter for KNeighbors classifier; n_neighbors: Number of neighbors to use for KNeighbors; p: Power parameter for the Minkowski metric in KNeighbors; weights: Weight function used in prediction for KNeighbors; max_features: Number of features to consider when looking for best split in Gradient Boosting; subsample: Fraction of samples used for fitting the individual base learners (Gradient Boosting); var_smoothing: Portion of the largest variance of all features added to variances for stability (GaussianNB); activation: Activation function for MLP; hidden_layer_sizes: Number of neurons in the ith hidden layer for MLP; max_iter: Maximum number of iterations (MLP); gamma: Kernel coefficient for SVM.  *Abbreviations:* MASLD: Metabolic dysfunction-associated steatotic liver disease, SGD Classifier: Stochastic Gradient Descent Classifier, GaussianNB: Gaussian Naive Bayes, MLP: Multi-Layer Perceptron Classifier | | |

| **Supplementary Table 4. Optimal hyperparameters of machine learning models for multiclass classification of steatosis severity (no MASLD vs. mild MASLD vs. moderate to severe MASLD) in the overall population and sex-specific subgroups.** | | |
| --- | --- | --- |
| **ML algorithms** | **Optimal Hyperparameter** | |
| Overall | |  |
| Random Forest | max_depth=10, min_samples_split=2, n_estimators=200 | |
| SGD Classifier | alpha=0.001, eta0=0.01, learning_rate='constant', loss='log_loss', penalty='elasticnet' | |
| Logistic Regression | C=0.1, penalty='l2', solver='lbfgs' | |
| KNeighbors Classifier | leaf_size=20, n_neighbors=10, p=1, weights='distance' | |
| Gradient Boosting | learning_rate=0.01, max_depth=4, max_features='sqrt', n_estimators=100, subsample=0.8 | |
| GaussianNB | var_smoothing=1e-09 | |
| MLP Classifier | activation='relu', alpha=0.0001, hidden_layer_sizes=(100, 100), solver='adam' | |
| Support Vector Machine | C=0.1, gamma='scale', kernel='linear' | |
|  |  | |
| Men |  | |
| Random Forest | max_depth=None, min_samples_split=10, n_estimators=100 | |
| SGD Classifier | alpha=0.001, eta0=0.01, learning_rate='constant', loss='log_loss', penalty='elasticnet' | |
| Logistic Regression | C=0.01, penalty='l2', solver='lbfgs' | |
| KNeighbors Classifier | leaf_size=20, n_neighbors=10, p=2, weights='distance' | |
| Gradient Boosting | learning_rate=0.01, max_depth=3, max_features='sqrt', n_estimators=200, subsample=0.8 | |
| GaussianNB | var_smoothing=1e-08 | |
| MLP Classifier | activation='tanh', alpha=0.0001, hidden_layer_sizes=(100, 100), solver='adam' | |
| Support Vector Machine | C=0.1, gamma='scale', kernel='linear' | |
|  |  | |
| Women |  | |
| Random Forest | max_depth=10, min_samples_split=2, n_estimators=500 | |
| SGD Classifier | alpha=0.001, eta0=0.01, learning_rate='constant', loss='log_loss', penalty='elasticnet' | |
| Logistic Regression | C=0.01, penalty='l2', solver='lbfgs' | |
| KNeighbors Classifier | leaf_size=20, n_neighbors=10, p=2, weights='distance' | |
| Gradient Boosting | learning_rate=0.01, max_depth=5, max_features='sqrt', n_estimators=100, subsample=1.0 | |
| GaussianNB | var_smoothing=1e-09 | |
| MLP Classifier | activation='relu', alpha=0.01, hidden_layer_sizes=(50,), solver='lbfgs' | |
| Support Vector Machine | C=0.1, gamma='scale', kernel='poly' | |
| ***Hyperparameter explanation:*** max_depth: Maximum depth of the tree; min_samples_split: Minimum number of samples required to split an internal node; n_estimators: Number of trees in the forest or boosting stages; alpha: Regularization strength for SGD and MLP classifiers; eta0: Initial learning rate for SGD; learning_rate: Learning rate schedule for weight updates (SGD); loss: Loss function used by the classifier; penalty: Regularization method; C: Inverse of regularization strength for Logistic Regression and SVM; solver: Algorithm to use in optimization; leaf_size: Leaf size parameter for KNeighbors classifier; n_neighbors: Number of neighbors to use for KNeighbors; p: Power parameter for the Minkowski metric in KNeighbors; weights: Weight function used in prediction for KNeighbors; max_features: Number of features to consider when looking for best split in Gradient Boosting; subsample: Fraction of samples used for fitting the individual base learners (Gradient Boosting); var_smoothing: Portion of the largest variance of all features added to variances for stability (GaussianNB); activation: Activation function for MLP; hidden_layer_sizes: Number of neurons in the ith hidden layer for MLP; max_iter: Maximum number of iterations (MLP); gamma: Kernel coefficient for SVM.  *Abbreviations:* MASLD: Metabolic dysfunction-associated steatotic liver disease, SGD Classifier: Stochastic Gradient Descent Classifier, GaussianNB: Gaussian Naive Bayes, MLP: Multi-Layer Perceptron Classifier | | |

| **Supplementary Table 5.** **Performance metrics of machine learning models for binary classification of MASLD presence (no MASLD vs. any degree MASLD) on the test set in the overall population and sex-specific subgroups.** | | | | | | | |
| --- | --- | --- | --- | --- | --- | --- | --- |
| **Model** | **AUC (95%CI)** | **F1 Score (95%CI)** | **Sensitivity (95%CI)** | | **Specificity (95%CI)** | **NPV (95%CI)** | **PPV (95%CI)** |
| Overall |  |  | |  |  |  |  |
| RF | 0.769 (0.694–0.843) | 0.856 (0.798–0.905) | | 0.906 (0.840–0.963) | 0.638 (0.500–0.772) | 0.794 (0.667–0.917) | 0.809 (0.736–0.885) |
| GNB | 0.690 (0.603–0.771) | 0.756 (0.683–0.822) | | 0.730 (0.624–0.818) | 0.650 (0.509–0.784) | 0.582 (0.446–0.710) | 0.785 (0.689–0.869) |
| SGD | 0.750 (0.673–0.827) | 0.826 (0.761–0.883) | | 0.848 (0.762–0.919) | 0.652 (0.521–0.783) | 0.710 (0.568–0.840) | 0.811 (0.726–0.885) |
| LR | 0.762 (0.690–0.832) | 0.833 (0.767–0.889) | | 0.847 (0.764–0.920) | 0.676 (0.533–0.804) | 0.715 (0.585–0.837) | 0.818 (0.732–0.893) |
| GBC | 0.758 (0.679–0.830) | 0.841 (0.781–0.894) | | 0.882 (0.810–0.944) | 0.629 (0.489–0.760) | 0.755 (0.629–0.875) | 0.806 (0.724–0.883) |
| MLP | 0.723 (0.637–0.797) | 0.802 (0.732–0.865) | | 0.811 (0.724–0.892) | 0.634 (0.491–0.769) | 0.660 (0.523–0.791) | 0.790 (0.701–0.872) |
| KNN | 0.700 (0.627–0.769) | 0.847 (0.786–0.898) | | 0.976 (0.938–0.999) | 0.430 (0.294–0.569) | 0.912 (0.778–0.999) | 0.747 (0.664–0.825) |
| SVM | 0.745 (0.674–0.821) | 0.829 (0.768–0.882) | | 0.858 (0.787–0.928) | 0.630 (0.500–0.766) | 0.722 (0.591–0.853) | 0.803 (0.716–0.881) |
|  |  |  | |  |  |  |  |
| Men |  |  | |  |  |  |  |
| RF | 0.753 (0.630–0.872) | 0.835 (0.730–0.921) | | 0.840 (0.703–0.949) | 0.663 (0.429–0.875) | 0.667 (0.421–0.889) | 0.843 (0.718–0.950) |
| GNB | 0.636 (0.566–0.708) | 0.417 (0.222–0.582) | | 0.270 (0.143–0.429) | 1.000 (1.000–0.999) | 0.395 (0.255–0.544) | 1.000 (1.000–0.999) |
| SGD | 0.794 (0.670–0.905) | 0.883 (0.805–0.951) | | 0.920 (0.820–0.999) | 0.664 (0.421–0.875) | 0.800 (0.583–0.999) | 0.850 (0.732–0.950) |
| LR | 0.791 (0.668–0.911) | 0.882 (0.794–0.951) | | 0.918 (0.818–0.999) | 0.676 (0.435–0.889) | 0.797 (0.562–0.999) | 0.850 (0.743–0.951) |
| GBC | 0.793 (0.680–0.904) | 0.897 (0.815–0.964) | | 0.973 (0.912–0.999) | 0.608 (0.375–0.846) | 0.916 (0.714–0.999) | 0.834 (0.714–0.932) |
| MLP | 0.862 (0.767–0.950) | 0.882 (0.795–0.953) | | 0.838 (0.714–0.946) | 0.887 (0.722–0.999) | 0.724 (0.524–0.905) | 0.940 (0.844–0.999) |
| KNN | 0.737 (0.620–0.857) | 0.877 (0.789–0.944) | | 0.975 (0.917–0.999) | 0.496 (0.250–0.727) | 0.899 (0.667–0.999) | 0.798 (0.674–0.907) |
| SVM | 0.793 (0.667–0.904) | 0.881 (0.795–0.951) | | 0.922 (0.829–0.999) | 0.665 (0.438–0.882) | 0.798 (0.571–0.999) | 0.851 (0.732–0.951) |
|  |  |  | |  |  |  |  |
| Women |  |  | |  |  |  |  |
| RF | 0.628 (0.527–0.730) | 0.743 (0.644–0.832) | | 0.794 (0.673–0.896) | 0.455 (0.286–0.630) | 0.580 (0.375–0.765) | 0.695 (0.580–0.815) |
| GNB | 0.609 (0.530–0.688) | 0.427 (0.276–0.565) | | 0.290 (0.167–0.420) | 0.938 (0.839–0.999) | 0.453 (0.333–0.582) | 0.875 (0.692–0.999) |
| SGD | 0.683 (0.587–0.781) | 0.775 (0.681–0.857) | | 0.816 (0.704–0.922) | 0.546 (0.379–0.720) | 0.651 (0.462–0.824) | 0.742 (0.623–0.857) |
| LR | 0.681 (0.582–0.781) | 0.794 (0.700–0.873) | | 0.877 (0.782–0.962) | 0.482 (0.310–0.667) | 0.710 (0.500–0.900) | 0.728 (0.615–0.836) |
| GBC | 0.631 (0.535–0.731) | 0.753 (0.659–0.835) | | 0.818 (0.696–0.918) | 0.450 (0.269–0.625) | 0.605 (0.385–0.810) | 0.701 (0.579–0.815) |
| MLP | 0.641 (0.523–0.750) | 0.725 (0.617–0.819) | | 0.735 (0.608–0.849) | 0.549 (0.375–0.724) | 0.563 (0.385–0.731) | 0.721 (0.588–0.851) |
| KNN | 0.611 (0.521–0.712) | 0.771 (0.679–0.850) | | 0.896 (0.804–0.978) | 0.324 (0.174–0.486) | 0.663 (0.411–0.889) | 0.675 (0.557–0.791) |
| SVM | 0.675 (0.569–0.781) | 0.779 (0.686–0.855) | | 0.835 (0.727–0.930) | 0.517 (0.333–0.700) | 0.665 (0.467–0.864) | 0.732 (0.611–0.848) |
| *Abbreviations:* MASLD: Metabolic dysfunction-associated steatotic liver disease, RF: Random Forest, SGD: Stochastic Gradient Descent Classifier, LR: Logistic Regression, KNN: K-Nearest Neighbors Classifier, GBC: Gradient Boosting Classifier, GNB: Gaussian Naive Bayes, MLP: Multi-Layer Perceptron Classifier, SVM: Support Vector Machine, AUC: Area Under the Receiver Operating Characteristic Curve, CI: Confidence Interval, F1 Score: Harmonic Mean of Precision and Recall, Sensitivity: True Positive Rate, Specificity: True Negative Rate, NPV: Negative Predictive Value, PPV: Positive Predictive Value. | | | | | | | |

| **Supplementary Table 6.** **Weighted performance metrics of machine learning models for multiclass classification of steatosis severity (no MASLD vs. mild MASLD vs. moderate to severe MASLD) on the test set in the overall population and sex-specific subgroups.** | | | | | | |
| --- | --- | --- | --- | --- | --- | --- |
| **Model** | **AUC (95%CI)** | **F1 Score (95%CI)** | **Sensitivity (95%CI)** | **Specificity (95%CI)** | **NPV (95%CI)** | **PPV (95%CI)** |
| Overall |  |  |  |  |  |  |
| RF | 0.762 (0.695–0.824) | 0.564 (0.472–0.650) | 0.590 (0.507–0.672) | 0.750 (0.693–0.800) | 0.578 (0.477–0.675) | 0.785 (0.726–0.837) |
| GNB | 0.725 (0.648–0.790) | 0.487 (0.399–0.565) | 0.490 (0.403–0.567) | 0.719 (0.657–0.773) | 0.491 (0.401–0.575) | 0.713 (0.649–0.765) |
| SGD | 0.739 (0.669–0.802) | 0.560 (0.476–0.645) | 0.566 (0.485–0.649) | 0.755 (0.697–0.805) | 0.563 (0.478–0.648) | 0.761 (0.697–0.814) |
| LR | 0.747 (0.673–0.808) | 0.590 (0.500–0.667) | 0.596 (0.507–0.672) | 0.758 (0.698–0.813) | 0.604 (0.514–0.690) | 0.775 (0.713–0.827) |
| GBC | 0.769 (0.702–0.831) | 0.573 (0.484–0.665) | 0.603 (0.522–0.687) | 0.750 (0.686–0.805) | 0.612 (0.508–0.709) | 0.794 (0.735–0.848) |
| MLP | 0.761 (0.696–0.830) | 0.671 (0.597–0.750) | 0.670 (0.597–0.746) | 0.811 (0.756–0.862) | 0.678 (0.602–0.755) | 0.812 (0.756–0.862) |
| KNN | 0.724 (0.656–0.787) | 0.527 (0.436–0.612) | 0.539 (0.455–0.619) | 0.710 (0.645–0.769) | 0.568 (0.471–0.654) | 0.738 (0.672–0.794) |
| SVM | 0.755 (0.687–0.817) | 0.583 (0.490–0.666) | 0.603 (0.515–0.679) | 0.746 (0.686–0.803) | 0.637 (0.533–0.726) | 0.785 (0.722–0.837) |
|  |  |  |  |  |  |  |
| Men |  |  |  |  |  |  |
| RF | 0.739 (0.638–0.834) | 0.490 (0.352–0.629) | 0.510 (0.382–0.636) | 0.741 (0.663–0.808) | 0.504 (0.358–0.647) | 0.744 (0.643–0.831) |
| GNB | 0.751 (0.655–0.838) | 0.234 (0.115–0.379) | 0.365 (0.236–0.509) | 0.695 (0.595–0.780) | 0.221 (0.087–0.405) | 0.757 (0.652–0.848) |
| SGD | 0.706 (0.584–0.815) | 0.551 (0.416–0.686) | 0.561 (0.436–0.691) | 0.775 (0.699–0.845) | 0.567 (0.426–0.708) | 0.765 (0.668–0.850) |
| LR | 0.748 (0.646–0.848) | 0.586 (0.444–0.725) | 0.619 (0.491–0.745) | 0.762 (0.655–0.848) | 0.671 (0.471–0.808) | 0.808 (0.711–0.890) |
| GBC | 0.777 (0.672–0.866) | 0.568 (0.435–0.702) | 0.583 (0.455–0.709) | 0.764 (0.680–0.837) | 0.612 (0.461–0.743) | 0.776 (0.681–0.854) |
| MLP | 0.723 (0.595–0.837) | 0.608 (0.468–0.742) | 0.616 (0.491–0.745) | 0.803 (0.724–0.874) | 0.625 (0.482–0.760) | 0.795 (0.697–0.873) |
| KNN | 0.717 (0.599–0.824) | 0.533 (0.393–0.666) | 0.548 (0.418–0.673) | 0.764 (0.680–0.837) | 0.545 (0.393–0.680) | 0.761 (0.653–0.845) |
| SVM | 0.753 (0.641–0.849) | 0.607 (0.475–0.739) | 0.616 (0.491–0.745) | 0.789 (0.712–0.860) | 0.633 (0.493–0.760) | 0.793 (0.703–0.873) |
|  |  |  |  |  |  |  |
| Women |  |  |  |  |  |  |
| RF | 0.706 (0.608–0.796) | 0.549 (0.435–0.665) | 0.564 (0.463–0.675) | 0.707 (0.617–0.788) | 0.586 (0.465–0.705) | 0.740 (0.651–0.824) |
| GNB | 0.684 (0.582–0.772) | 0.348 (0.236–0.477) | 0.462 (0.350–0.575) | 0.769 (0.711–0.819) | 0.286 (0.180–0.411) | 0.731 (0.637–0.812) |
| SGD | 0.655 (0.559–0.747) | 0.471 (0.353–0.580) | 0.489 (0.375–0.600) | 0.664 (0.577–0.742) | 0.482 (0.352–0.609) | 0.686 (0.590–0.770) |
| LR | 0.702 (0.608–0.786) | 0.495 (0.386–0.613) | 0.552 (0.450–0.662) | 0.681 (0.595–0.759) | 0.465 (0.350–0.584) | 0.741 (0.650–0.827) |
| GBC | 0.698 (0.599–0.789) | 0.515 (0.397–0.630) | 0.540 (0.438–0.650) | 0.676 (0.582–0.761) | 0.593 (0.449–0.714) | 0.723 (0.626–0.811) |
| MLP | 0.558 (0.454–0.661) | 0.421 (0.307–0.533) | 0.426 (0.312–0.537) | 0.650 (0.564–0.732) | 0.433 (0.319–0.553) | 0.650 (0.559–0.736) |
| KNN | 0.712 (0.606–0.805) | 0.513 (0.382–0.635) | 0.541 (0.425–0.650) | 0.676 (0.586–0.760) | 0.600 (0.456–0.715) | 0.729 (0.634–0.815) |
| SVM | 0.718 (0.630–0.798) | 0.571 (0.448–0.685) | 0.603 (0.487–0.713) | 0.724 (0.644–0.796) | 0.624 (0.458–0.753) | 0.775 (0.691–0.856) |
| *Abbreviations:* MASLD: Metabolic dysfunction-associated steatotic liver disease, RF: Random Forest, SGD: Stochastic Gradient Descent Classifier, LR: Logistic Regression, KNN: K-Nearest Neighbors Classifier, GBC: Gradient Boosting Classifier, GNB: Gaussian Naive Bayes, MLP: Multi-Layer Perceptron Classifier, SVM: Support Vector Machine, AUC: Area Under the Receiver Operating Characteristic Curve, CI: Confidence Interval, F1 Score: Harmonic Mean of Precision and Recall, Sensitivity: True Positive Rate, Specificity: True Negative Rate, NPV: Negative Predictive Value, PPV: Positive Predictive Value. | | | | | | |

| **Supplementary Table 7.** **Sensitivity analysis for top-performing machine learning models using stratified 5-fold cross-validation for predicting MASLD presence and steatosis severity in the overall population, in men, and in women.** | | | | | | |
| --- | --- | --- | --- | --- | --- | --- |
| **Model** | **AUC**  **(mean ± SD)** | **F1 Score**  **(mean ± SD)** | **Sensitivity**  **(mean ± SD)** | **Specificity**  **(mean ± SD)** | **NPV**  **(mean ± SD)** | **PPV**  **(mean ± SD)** |
| **Binary classification**  (no MASLD / any degree MASLD) | | | | | | |
| Overall (RF) | 0.828 ± 0.048 | 0.832 ± 0.032 | 0.873 ± 0.043 | 0.608 ± 0.093 | 0.736 ± 0.071 | 0.796 ± 0.043 |
| Men (GBC) | 0.866 ± 0.058 | 0.850 ± 0.038 | 0.909 ± 0.055 | 0.533 ± 0.139 | 0.755 ± 0.119 | 0.801 ± 0.051 |
| Women (LR) | 0.832 ± 0.081 | 0.807 ± 0.083 | 0.841 ± 0.123 | 0.626 ± 0.080 | 0.735 ± 0.163 | 0.780 ± 0.053 |
|  |  |  |  |  |  |  |
| **Multiclass classification**  (no MASLD / mild MASLD / moderate to severe MASLD) | | | | | | |
| Overall (MLP) | 0.696 ± 0.047 | 0.546 ± 0.045 | 0.547 ± 0.048 | 0.747 ± 0.026 | 0.750 ± 0.030 | 0.551 ± 0.046 |
| Men (MLP) | 0.702 ± 0.065 | 0.547 ± 0.070 | 0.555 ± 0.067 | 0.768 ± 0.035 | 0.769 ± 0.036 | 0.556 ± 0.069 |
| Women (SVM) | 0.753 ± 0.040 | 0.490 ± 0.057 | 0.560 ± 0.044 | 0.674 ± 0.029 | 0.819 ± 0.041 | 0.627 ± 0.075 |
| **Note:** All metrics for multiclass classification are reported as weighted averages based on class support.  *Abbreviations:* MASLD: Metabolic dysfunction-associated steatotic liver disease, RF: Random Forest, LR: Logistic Regression, MLP: Multi-Layer Perceptron Classifier, SVM: Support Vector Machine, AUC: Area Under the Receiver Operating Characteristic Curve, CI: Confidence Interval, F1 Score: Harmonic Mean of Precision and Recall, Sensitivity: True Positive Rate, Specificity: True Negative Rate, NPV: Negative Predictive Value, PPV: Positive Predictive Value. | | | | | | |
